# Family a GPCR heteromers in animal models

**DOI:** 10.3389/fphar.2014.00226

**Published:** 2014-10-09

**Authors:** Javier González-Maeso

**Affiliations:** Departments of Psychiatry and Neurology, Friedman Brain Institute, Icahn School of Medicine at Mount SinaiNew York, NY, USA

**Keywords:** G protein-coupled receptor (GPCR), dimerization, heteromer, heterocomplex, serotonin 5-HT_2A_ receptor, metabotropic glutamate 2 receptor, molecular pharmacology

## Introduction

G protein-coupled receptors (GPCRs) were assumed to exist and function in the plasma membrane as monomeric proteins that became activated by binding of one agonist ligand to one receptor molecule (Bourne et al., 1990). However, although previous findings based on rather indirect measures such as radioligand binding had suggested a direct interaction of two receptors with each other (Limbird et al., 1975; Ferre et al., 1991), it was the application of a protein-protein interaction assay by bioluminescence resonance energy transfer (BRET) that revealed the phenomenon of molecular proximity between beta2-adrenergic receptors in living cells (Angers et al., 2000). Since then, this topic has been a major subject of research and numerous in silico and *in vitro* studies have suggested expression of family A GPCRs as homodimers and higher-order homomers in heterologous expression systems. However, the demonstration that reconstitution of a single beta2-adrenergic receptor molecule into lipoprotein particles leads to efficient activation of G proteins raised concerns about the functional significance of family A GPCR homomers (Whorton et al., 2007), and this is currently a controversial topic (for an extensive review on GPCR homodimers/homomers, see Milligan, 2013; see also Bouvier and Hebert, 2014; Lambert and Javitch, 2014).

Another fundamental yet relatively independent question is that related to expression of different GPCR subtypes as heteromers. It is well accepted that the family C GABA_B_ receptor needs two protomers (GABA_B_-R1 and GABA_B_-R2) to reach the plasma membrane as a functional dimeric receptor (Jones et al., 1998; Kaupmann et al., 1998; White et al., 1998). On the other hand, although multiple lines of evidence indicate that family A GPCR heteromers may exist, particularly in tissue cultures (González-Maeso, 2011; Ferre et al., 2014), only relatively recent studies started to test this formulation in whole animal models.

## Family A GPCR heteromers in whole animal models

One of the main limitations of the classical techniques used to define GPCR heteromeric formation is the translation of findings obtained in cellulo into physiological or behavioral assays in whole animal models. In this context, co-immunoprecipitation is an approach commonly used to examine protein-protein interaction in native tissue (Milligan and Bouvier, 2005). GPCR antibodies are usually neither specific nor sensitive and therefore validation assays in knockout mice are often required (Fribourg et al., 2011; Moreno et al., 2012). Considering this, it is also clear that demonstration of co-immunoprecipitation in native tissues does not imply the existence of a heteromeric assembly, as they may form part of same protein complex through for example PDZ domain-binding motifs at the end of the C-terminal tails of both receptor types together with adaptor proteins (Magalhaes et al., 2010). Remarkably, there are only a few studies that have investigated GPCR heteromeric formation in living animals, and due to the lack of biophysical methods applicable to study protein-protein interactions in preclinical models, their experimental approaches were mostly focused on signaling and behavioral outcomes rather than on the existence of molecular proximity between different GPCR subtypes.

Although it does not measure molecular proximity, an attractive approach to define whether heteromeric formation is involved in behavioral phenotypes is the use of peptides that disrupt receptor complex formation. These peptides tested *in vivo* are usually selected according to findings previously obtained in heterologous expression systems. As an example, it was demonstrated that the G_s_-coupled dopamine D_1_ receptor and the G_i/o_-coupled dopamine D_2_ form a receptor complex that induces Ca^2+^ release via a G_q/11_-dependent pathway (Lee et al., 2004), and that the region of Met-257—Glu-271 (intracellular loop 3; D2_IL3-29-2_) but not Asn-243—Ile-256 (intracellular loop 3; D2_IL3-29-1_) of the dopamine D_2_ receptor can pull-down the dopamine D_1_ receptor. Based on a Tat-tagged peptide approach, it was shown that intracerebroventricular administration of the peptide D2_IL3-29-2_, which disrupts heteromeric formation between dopamine D_1_ and D_2_ receptors *in vitro*, induces antidepressant-like effects in rats (Pei et al., 2010). More recent findings using serial deletions and point mutations further demonstrate that dopamine D_1_ receptor carboxyl tail residues Glu-404 and Glu-405 are critical in mediating the interaction with the D_2_ receptor, and that administration of a disrupting peptide Tat-D1 modulates depression-like behavior in rats such as forced swim test (Hasbi et al., 2014). A similar approach was used to block the association as a GPCR heteromer between the mu-opioid receptor isoform MOR1D and the gastrin-releasing peptide receptor (GRPR) in the spinal cord (Liu et al., 2011). The authors demonstrated that the C-terminus of MOR1D is critical for MOR1D-GRPR heteromeric formation. Using a Tat-fusion peptide, they also found that a motif consisting of seven amino acids of the MOR1D C-terminus (RNEEPSS) attenuates morphine-induced scratching, but not morphine-induced analgesia.

The question of whether GPCR heteromers exist *ex vivo* has been addressed using time-resolved Förster resonance energy transfer FRET (TR-FRET) in plasma membrane preparations of mouse brain. It was found that the dopamine D_2_ receptor and the ghrelin receptor (GHSR1a) co-localize in mouse striatum, hippocampus and hypothalamus (Kern et al., 2012). When membrane preparations from hypothalamus were incubated with red-ghrelin (acceptor fluorophore) and an anti-D_2_ receptor antibody together with a europium cryptate-labeled secondary antibody (donor fluorophore), a significantly TR-FRET signal was observed. Although TR-FRET signal is eliminated in hypothalamic membrane preparations of GHSR1a knockout mice, which supports specificity, these findings were observed *ex vivo* in plasma membrane preparations and further investigation will be necessary to confirm the existence of GHSR1a-D_2_ heteromeric formation in hypothalamus *in vivo*.

Another indirect approach to test whether GPCR heteromeric formation affects behavioral phenotypes is the use of chimeric constructs that according to biophysical assays in tissue culture do not form heteromeric complexes. Examples include the 5-HT_2A_-mGlu2 heteromeric receptor complex (Gonzalez-Maeso et al., 2008) and the MT_1_-MT_2_ melatonin heteromeric receptor complex (Baba et al., 2013). Serotonin 5-HT_2A_ and metabotropic glutamate 2 (mGlu2) receptors have been shown to form a GPCR heteromeric complex in HEK293 cells. Using chimeric constructs, it was demonstrated that three residues located at the intracellular end of TM4 of mGlu2 are necessary to form a complex with the 5-HT_2A_ receptor (Ala-677^4.40^, Ala-681^4.44^, Ala-685^4.48^) (Fribourg et al., 2011; Moreno et al., 2012). Head-twitch is a rodent behavior model induced by hallucinogenic 5-HT_2A_ agonist such as lysergic acid diethylamide (LSD) and DOI (Hanks and Gonzalez-Maeso, 2013). This behavior requires expression of 5-HT_2A_ receptor in cortical pyramidal neurons (Gonzalez-Maeso et al., 2007) and is absent in mGlu2 knockout mice (Moreno et al., 2011), which supports that mGlu2 is necessary for 5-HT_2A_-dependent behavioral events. Using a virally-mediated (HSV) over-expression approach, it was demonstrated that the head-twitch response induced by the hallucinogenic 5-HT_2A_ receptor agonist DOI was rescued in mGlu2 knockout mice over-expressing wild-type mGlu2 in frontal cortex, and that this did not occur in mGlu2 knockout mice over-expressing mGlu2deltaTM4N—a mGlu2/mGlu3 chimeric construct that according to previous findings *in vitro* and in cellulo does not form the 5-HT_2A_-mGlu2 receptor heteromer (Moreno et al., 2012). A similar approach was used to investigate function of the MT_1_-MT_2_ melatonin heteromeric receptor complex *in vivo* in mouse (Baba et al., 2013). The electroretinogram (ERG), consisting mainly of an a-wave and a b-wave, is commonly used to assess retinal function. Using transgenic mice that express MT_2_-P95L (mutant that does not form the MT_1_-MT_2_ heteromeric receptor complex in HEK293 cells), it was shown that control mice responded to melatonin injection with an increase in the amplitude of the a-wave and b-wave, whereas MT_2_-P95L did not.

Although these events have been proposed to represent a demonstration of GPCR heteromeric expression, thereby suggesting a new target for drug design, their conclusions in animal models were based largely on indirect approaches that measured phenotypes affected by manipulations such as chimeric constructs or Tat-tagged peptides that impact heteromeric organization *in vitro*. Consequently, it remains unclear as to whether different GPCR subtypes exist in close molecular proximity *in vivo* in whole animal models, or alternatively if these phenotypes result of signaling mechanisms that are independent of GPCR heteromeric formation. Detailed measurement of such molecular proximity *in vivo*, as well as the processes that control GPCR heteromerization in whole animal models, will require further study.

## Limitations, future directions, and concluding remarks

Although a wealth of data from *in vitro* and in cellulo models have established the important role of GPCR heteromers in mediating precise and distinct roles in signaling cascades, their influence in the establishment of complex behavioral phenotypes remains to be fully elucidated. For instance, certain physiological and behavioral outcomes could conceivably be altered in the presence of peptides that according to *in vitro* or in cellulo assays disrupt GPCR heteromeric assembly. Similarly, the use of viral-mediated over-expression or transgenic animals could translate into animal models previous findings with receptor mutants that do not form heterocomplexes *in vitro* or in cellulo. However, a more precise understanding of such structural assembly obtained in rodent models will be necessary to fully define whether GPCR heteromers exist and function *in vivo*. Some of these strategies include the use of FRET (McGinty et al., 2011) of BRET (Dragulescu-Andrasi et al., 2011) imaging of protein-protein interactions in living mice.

An important challenge in the fields of GPCR research and molecular pharmacology is to develop an integrated understanding of how various mechanisms communicate with each other to ultimately orchestrate the formation of heteromeric complexes between some but not all GPCR subtypes. Potential mechanisms that are critical for this interaction specificity include specific pairs of residues that govern heteromeric formation, clustering of GPCRs in membrane microdomains, and crosstalk between receptors and a plethora of multidomain scaffolding proteins. Another important question to be addressed by future research is the molecular basis through which GPCR heteromers affect G protein function. For example, it has been shown that drugs that activate the G_q/11_-coupled 5-HT_2A_ receptor induce both G_q/11_- and G_i/o_-dependent signaling in HEK293 cells co-expressing 5-HT_2A_ and the G_i/o_-coupled mGlu2 receptor as a GPCR heteromer (Gonzalez-Maeso et al., 2008). Although findings in knockout mice suggest that co-expression of 5-HT_2A_ and mGlu2 receptors is necessary to activate G_q/11_ and G_i/o_ by 5-HT_2A_ agonists in mouse frontal cortex membrane preparations (Fribourg et al., 2011), whether heteromeric formation is needed in living mice for this signaling crosstalk remains unknown. Similarly, more work is required both in cellulo and in animal models to solve whether G_q/11_ and G_i/o_ simultaneously or sequentially couple to the 5-HT_2A_-mGlu2 heteromeric receptor complex upon agonist binding to one of the two promoters.

Another significant limitation to our current understanding of GPCR heteromeric function is the lack of knowledge about physical stability of family A GPCR heteromers in animal models. Previous findings in HEK293 cells convincingly demonstrate that the alpha1B-adrenergic receptor forms higher-order oligomers, and that receptor oligomerization is required for receptor maturation and plasma membrane delivery (Lopez-Gimenez et al., 2007). On the other hand, results based on an experimental approach that recruits beta2-adrenergic receptors into artificial domains on the surface of living HEK293 cells suggest that the components of family A GPCR homomers interact transiently (Fonseca and Lambert, 2009; Gavalas et al., 2013). A similar conclusion has been reached using total internal reflection fluorescence microscopy (TIRFM) to visualize individual molecules in isolated CHO cells—the authors observed a transient association and dissociation of muscarinic M_1_ receptor dimers in real time (Hern et al., 2010). Much further work is needed to characterize where along the pathway from synthesis to maturation and degradation do GPCR heteromers form. It also remains uncertain the stability of family A GPCR heteromers both *in vitro* and in whole animal models. Many studies examining homomeric GPCR interfaces report that residues of both TM1 and TM4 form symmetrical interfaces that lead to higher order species in heterologous expression systems (Guo et al., 2005, 2008), and this has been supported further by a number of recent crystal structures (Wu et al., 2012; Huang et al., 2013). However, it remains to be fully elucidated whether different homomeric and heteromeric organizations (e.g., squares and/or parallelograms) might exist in native tissue. These are all key questions that require further technical advances.

In conclusion, although a range of approaches has been applied and this has led to a general appreciation that GPCR heteromers affect receptor trafficking, pharmacology and function in cellulo, much more work is needed to probe the role of GPCR heteromerization *in vivo*. These advances in GPCR heteromeric research are now occurring at a rapid pace and promise to greatly contribute to the future of molecular medicine.

### Conflict of interest statement

The author declares that the research was conducted in the absence of any commercial or financial relationships that could be construed as a potential conflict of interest.
